# Metabolic control of porin permeability influences antibiotic resistance in *Escherichia coli*

**DOI:** 10.1038/s41564-025-02175-5

**Published:** 2025-11-24

**Authors:** Santiago E. Caño Muñiz, Stephen Trigg, Georgeos Hardo, Anja Hagting, Ieuan E. Evans, Christopher Ruis, Ali F. Alsulami, David Summers, Felicity Crawshay-Williams, Tom L. Blundell, Lucas Boeck, Somenath Bakshi, R. Andres Floto

**Affiliations:** 1 The Victor Phillip Dahdaleh Heart and Lung Research Institute, University of Cambridge, Cambridge, UK; 2 University of Cambridge Molecular Immunity Unit, Department of Medicine, MRC Laboratory of Molecular Biology, Cambridge, UK; 3 Department of Biomedicine, University of Basel, Basel, Switzerland; 4 Department of Engineering, University of Cambridge, Cambridge, UK; 5 Department of Biology, United Arab Emirates University, Al Ain, United Arab Emirates; 6 Cambridge Centre for Lung Infection, Royal Papworth Hospital, Cambridge, UK; 7 Cambridge Centre for AI in Medicine, Cambridge, UK; 8 Department of Biochemistry, University of Cambridge, Cambridge, UK; 9 Department of Biochemistry, Faculty of Science, King Abdulaziz University, Jeddah, Saudi Arabia; 10 Department of Genetics, University of Cambridge, Cambridge, UK

**Keywords:** Antibiotics, Bacteriology

## Abstract

Porins mediate the passage of hydrophilic nutrients and antibiotics across the outer membrane but might contribute to proton leak from the periplasm, suggesting that their conductance could be regulated. Here we show, using single-cell imaging, that porin permeability in *Escherichia coli* is controlled by changes in periplasmic H^+^ and K^+^ concentration. Conductance through porins increases with low periplasmic H^+^ caused by starvation, promoting nutrient uptake, and decreases with periplasmic acidification during growth in lipid media, limiting proton loss. High metabolic activity during growth in glucose media, however, activates the inner membrane voltage-gated potassium channel, Kch, increasing periplasmic potassium and enhancing porin permeability to dissipate reactive oxygen species. This metabolic control of porin permeability explains the observed increase in ciprofloxacin resistance of bacteria catabolizing lipids and clarifies the impact of mutations in central metabolism genes on drug resistance, identifying Kch as a therapeutic target to improve bacterial killing by antibiotics.

## Abstract (web summary)

The permeability of bacterial porins is dynamically regulated by periplasmic proton and potassium concentrations, altering antibiotic resistance.

## Main

The outer membrane of Gram-negative bacteria forms a physical and mechanical barrier that protects them from chemical and biological attacks^1–3^. Bacterial porins are water-filled β barrel channels across this membrane^4^, mediating permeability to nutrients, such as glucose^5,6^, and to many antibiotics^7–9^, including β lactams^10–12^, carbapenems^13,14^ and fluoroquinolones^15–17^.

However, porins might also contribute to proton leakage from the periplasm^18,19^, by means of conventional diffusion or the Grotthuss mechanism through water-filled pores (although this has not been experimentally demonstrated to date), thereby dissipating the proton motive force generated by the electron transport chain (ETC) that is required for ATP synthesis via oxidative phosphorylation^20–24^ and other cellular processes^22^, such as active solute transport, drug efflux^25^ and flagellar motion^26–28^.

We, therefore, wondered whether the conductance through bacterial porins might be dynamically controlled to balance nutrient uptake and energy production and consequently regulate antibiotic permeability and, hence, resistance. While previous studies, predominantly using lipid bilayer electrophysiological measurements of purified porins^13,17,29–31^, have suggested the potential for regulation or gating of porin conductance through voltage and/or monovalent cations^32–35^, their physiological relevance and activation mechanisms remain unclear.

We, therefore, sought to examine the control of porin permeability in situ using real-time fluorescence imaging of individual bacteria encoding fluorescence sensors and optogenetic probes. In this Article, we demonstrate that porin permeability in *Escherichia coli* is dynamically regulated by changes in periplasmic H^+^ and K^+^ concentrations caused by the activity of the ETC and the inner membrane voltage-gated potassium channel Kch. This ionic regulation explains how bacteria balance nutrient uptake with energy conservation and provides a mechanistic basis for the metabolic control of antibiotic resistance.

## Results and discussion

### Porin permeability is regulated by periplasmic H^+^ and K^+^ ions

**Fig. 1:**
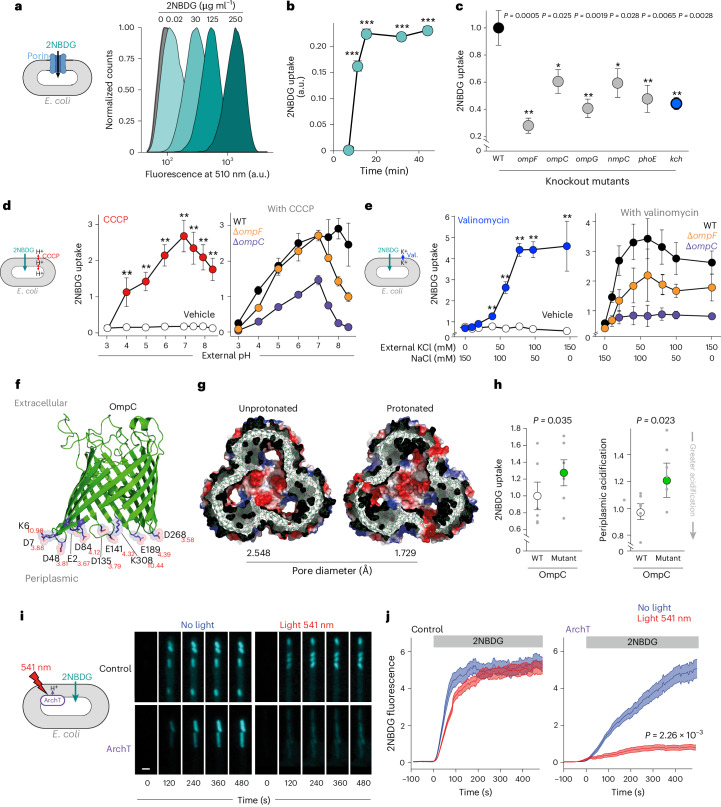
Bacterial porins are regulated by changes in internal proton and potassium concentrations. **a**,**b**, Accumulation of the fluorescent glucose analogue 2NBDG into *E. coli* following 10 min incubation at a range of concentrations (**a**) and at 20 μg ml^−1^ over a range of incubation times (**b**), showing significant 2NBDG uptake at 5 min (*P* = 2.54 × 10^−5^), 10 min (*P* = 2.53 × 10^−5^), 30 min (*P* = 2.47 × 10^−5^) and 45 min (*P* = 3.55 × 10^−5^); (*n* ≥ 10 biological replicates for each condition). **c**, Accumulation at 10 min of 20 μg ml^−1^ 2NBDG in WT *E. coli* (black) or isogenic knockout strains for the major porins *ompF* and *ompC*, minor porins *ompG*, *nmpC* and *phoE* (grey), and the voltage-gated potassium channel *kch* (blue; *n* = 8 biological replicates for each condition). **d**, Effect on 2NBDG accumulation (left) in WT *E. coli* of changing external pH alone (white) or in the presence of CCCP (250 μM; red) which led to significantly increased permeability at pH 3 (*P* = 2.61 × 10^−3^), pH 4 (*P* = 8.86 × 10^−9^), pH 5 (*P* = 8.86 × 10^−9^), pH 6 (*P* = 1.42 × 10^−8^) and pH 7–8.5 (all *P* ≤ 8.86 × 10^−9^); and (right) in WT (black), Δ*ompF* (yellow) and Δ*ompC* (purple) *E. coli* in the presence of CCCP (*n* = 12 biological replicates for each condition). **e**, Effect on 2NBDG accumulation (left) in WT *E. coli* of changing external potassium concentrations (while maintaining monovalent cations constant) alone (white) or in the presence of valinomycin (100 μM; blue) which caused significant increase in permeability at 10 mM (*P* = 7.82 × 10^−2^), 20 mM (*P* = 3.24 × 10^−2^), 40 mM (*P* = 5.91 × 10^−3^), 60 mM (*P* = 6.19 × 10^−3^), 80 mM (*P* = 2.18 × 10^−3^), 100 mM (*P* = 3.06 × 10^−3^) and 150 mM (*P* = 2.76 × 10^−2^) external K^+^; and (right) in WT (black), Δ*ompF* (yellow) and Δ*ompG* (purple) *E. coli* in the presence of valinomycin (*n* = 3 biological replicates for each condition). *y* axes in **d** and **e** show normalised 2NBDG fluorescence. **f**, Structure of OmpC (PDB 2J1N) highlighting periplasmic residues (E2, E43, E189, K6, K308, D7, D48, D135, D141, D268) likely to be affected by periplasmic acidification (their −log_10_ acid dissociation constants (pKa) are shown in red). **g**, Cross-sectional views of molecular dynamic simulations of the effect of protonation of residues shown in **f** on pore diameter, thereby modelling the potential impact of periplasmic acidification. The structure shown is the average of sampled conformers. **h**, The effect on porin permeability (assessed using 2NBDG uptake (shown as 2NBDG fluorescence normalised to wild type); left) and on periplasmic pH (measured using a pHuji-based fluorescent reporter (shown as pHuji fluorescence normalised to wild type); right) of expressing a mutant OmpC where all charged residues on the periplasmic surface of OmpC were replaced with alanines (E23A, K27A, D28A, E64A, D69A, D156A, D162A, D289A, K329A); *Mutant OmpC* (green) compared to isogenic WT control (white). **i**,**j**, Single-cell fluorescence imaging of *E. coli* for a single experiment grown in a microfluidic perfusion system (mother machine^55^) shows 2NBDG accumulation over time in WT bacteria expressing empty vector (WT) or expressing the light-activated proton pump ArchT in the inner membrane in the presence (blue) or absence (red) of 541 nm light exposure showing representative images (**i**) and quantification (shown as background-corrected 2NBDG fluorescence in a.u.) (**j**). Scale bar, 1 μm. Data (mean ± s.e.m.) are representative of at least three independent experiments performed at least in triplicate, imaging at least 50 individual bacteria per condition on each occasion. All data (mean ± s.e.m.) are representative of at least three independent experiments performed at least in triplicate. *P < 0.05, **P < 0.01, ***P < 0.001 (two-sided Student’s *t*-test). Source data

**Extended Data Fig. 1:**
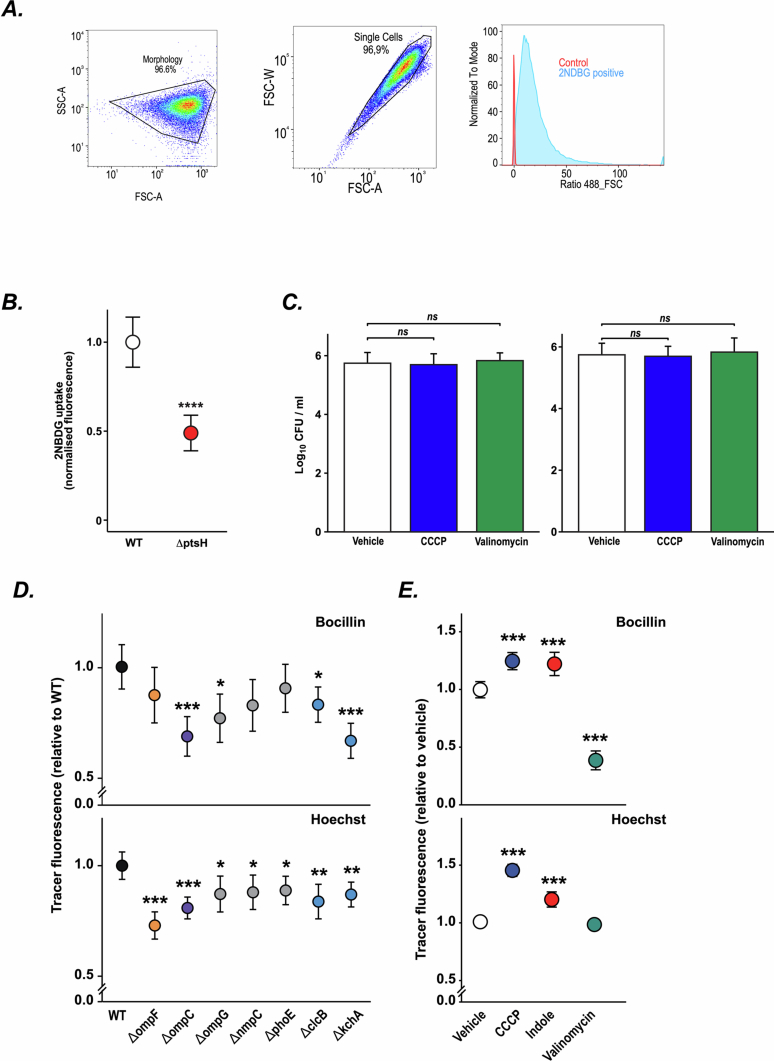
Effect of treatments and gene deletions on porin permeability and viability in *E. coli.* (**A**) Uptake of 2NBDG (quantified by flow cytometry) by wild type E. coli. Gating strategy shown. (**B**) Uptake of 2NBDG by wild type (WT; white) and ΔptsH (red) *E. coli*. (**C**) Effect of CCCP or Valinomycin on E. coli viability measured in colony-forming units (CFU) per ml under conditions identical to those shown in Fig. 1e. Cells were grown in glucose (left) or lipid-based media (right) and treated with vehicle (white), CCCP (250 μM; blue), or Valinomycin (100 μM; green) for 30 minutes. (**D**) Accumulation of bocillin (0.5 µg/mL; top) or Hoechst (10 µg/mL: bottom) in E. coli (quantified by flow cytometry) following 10 min incubation using wild type bacteria (WT; black) or isogenic knockouts for ompF (yellow), ompC (purple), minor porins (ompG, nmpC, phoE; grey), and the ion channels clcB and kch (blue). (**E**) Accumulation of bocillin (0.5 µg/mL; top) or Hoechst (10 µg/mL: bottom) in wild type *E. coli* (quantified by flow cytometry) following treatment with the protonophores CCCP (blue) or Indole (red) or the potassium ionophore valinomycin (green), normalised to vehicle alone (white). Data (mean ± SEM) are representative of at least three independent experiments performed in at least triplicate. *p < 0.05, ** p < 0.01, *** p < 0.001 (Student’s t-test). Source data

We first used the fluorescent glucose analogue 2-deoxy-2-[(7-nitro-2,1,3-benzoxadiazol-4-yl)amino]-D-glucose (2NBDG), whose entry into *E. coli* is concentration (Fig. 1a) and time dependent (Fig. 1b) and known to be mediated by porins^36–40^, to screen bacterial knockout mutants from the KEIO collection^41^ for altered porin permeability. As expected, we observed that 2NBDG accumulation, quantified by flow cytometry, was reduced in bacterial mutants of porins (*ompF*, *ompC*, *ompG*, *nmpC* and *phoE*) and the inner membrane glucose transporter *ptsH* (Extended Data Fig. 1) but surprisingly also in mutants for several ion channels, including the putative voltage-gated potassium channel *kch*^42^ (Fig. 1c), findings that were mirrored using other tracers of porin permeability (such as the fluorescent penicillin analogue Bocillin FL^43,44^ and Hoechst^45^; Extended Data Fig. 1).

To examine whether porin permeability might be under ionic regulation, we monitored 2NBDG uptake using flow cytometry in wild-type (WT) *E. coli* across a range of external pH. We found that 2NBDG uptake was significantly enhanced as external pH increased but only in the presence of the protonophore carbonyl cyanide m-chlorophenyl hydrazone (CCCP) (Fig. 1d). Porin permeability was also enhanced as external K^+^ concentrations increased (while maintaining total external monovalent cations constant) but only in the presence of the potassium ionophore valinomycin (Fig. 1e). Although CCCP and valinomycin may have several collateral effects on bacteria, these observations, which are not due to ionophore-mediated bacterial death (Extended Data Fig. 1), suggest that internal (rather than external) proton and potassium levels control porin permeability, effects which, based on examining knockout strains, are mediated predominantly by conductance changes of the two major porins, OmpC and OmpF (Fig. 1d,e).

**Extended Data Fig. 2:**
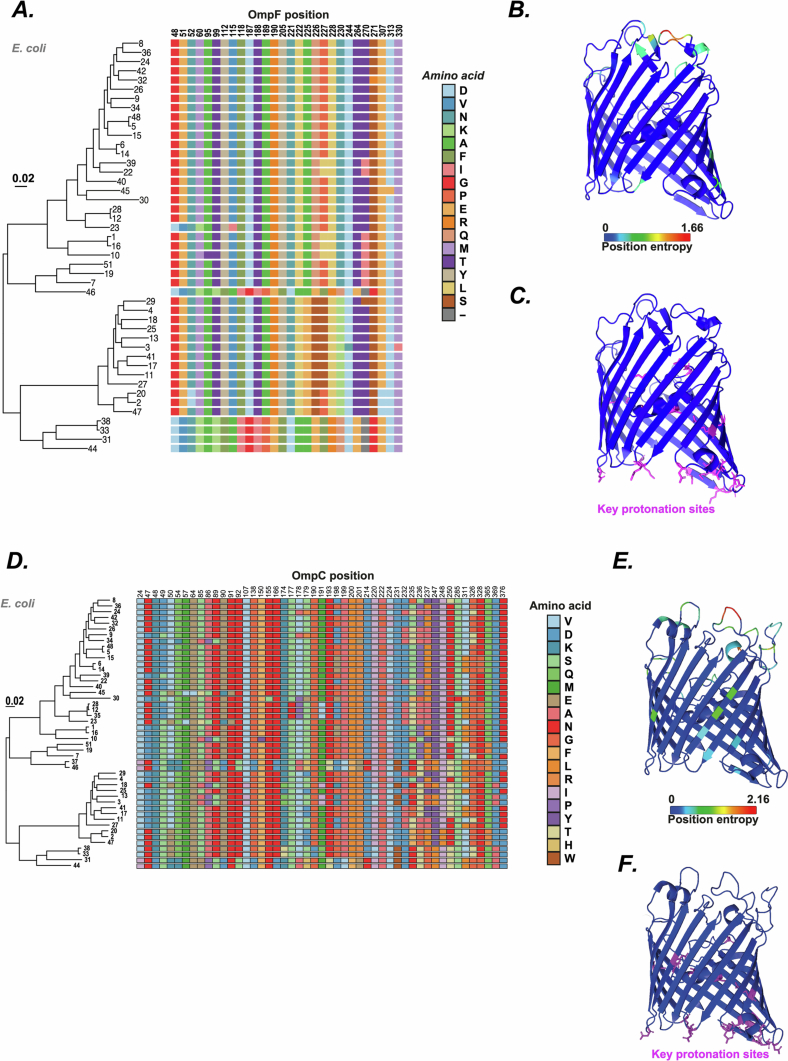
OmpF and OmpC mutations found across phylogenetic tree of *E. coli.* (**A**-**D**) Left Phylogenetic relationships between E. coli lineages based on core genes13. Scale bar shows expected substitutions per site. Right Amino acids at each variable position within OmpF (**A**) or OmpC (**D**) for each lineage. (**B** and **E**) OmpF (PDB accession 2OMF) or OmpC (PDB accession 2J1N) molecular structure coloured by position entropy; a measure of sequence variation at the position. (**C** and **F**) Same molecular structures coloured with key sites labelled (pink) that have been implicated by our molecular dynamics simulations as being involved in periplasmic ion-dependent regulation of pore conductance.

Periplasmic pH and K^+^ may directly affect the pore diameter of porins, as suggested by previous lipid bilayer electrophysiological studies that have reported pH-dependent and K^+^-dependent effects on porin conductance^7,29,30,46–49^. The periplasmic surfaces of both OmpC and OmpF are decorated with charged residues (Fig. 1f) that are phylogenetically conserved^50,51^ despite the frequent acquisition of other porin mutations (Extended Data Fig. 2) and could potentially be affected by changes in both periplasmic pH and K^+^ (via altered protonation and electrostatic shielding, respectively^49^).

**Extended Data Fig. 3:**
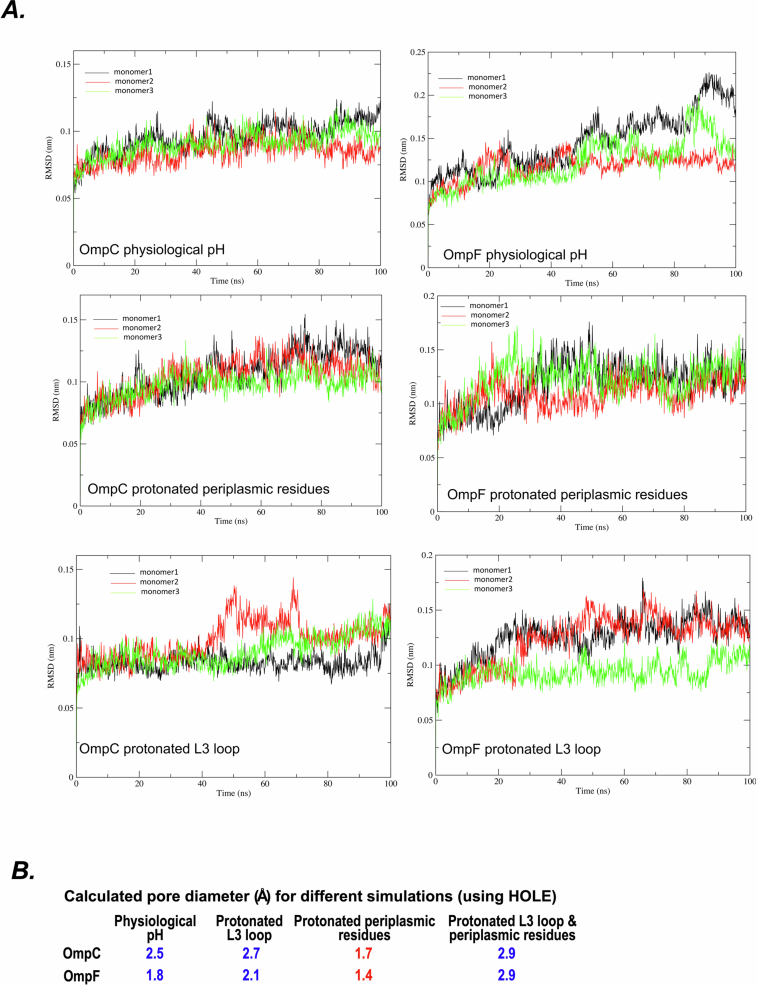
Molecular dynamic (MD)simulations of the change in porin pore size during acidification. (**A**) MD simulation of OmpC (left) and OmpF (right) trimers at (top) physiological pH, (middle) following protonation of periplasmic amino acid residues, and (bottom) following protonation of the L3 loop within the channel. (**B**) Calculated pore diameters (in angstroms) using HOLE software for a range of different simulation conditions.

**Extended Data Fig. 4:**
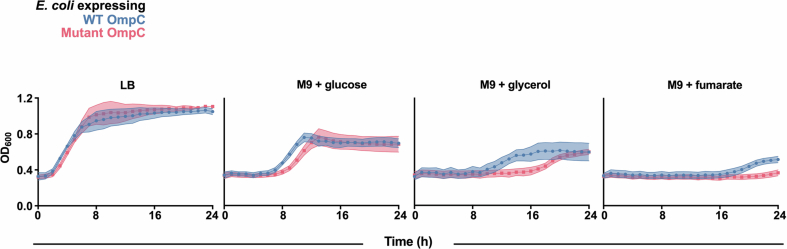
Growth dynamics of wild type and mutant OmpC-expressing *E. coli.* The effect on E. coli growth in a variety of different media of porin permeability (assessed using 2NBDG uptake; left) of expressing a mutant OmpC where all charged residues on the periplasmic surface of OmpC were replaced with alanines (E23A, K27A, D28A, E64A, D69A, D156A, D162A, D289A, K329A); Mutant OmpC; red) compared to isogenic wild type control (blue).

To further examine the potential mechanisms involved in the control of porin permeability by periplasmic pH, we first ran molecular dynamic simulations that suggested that protonation of amino acid residues on the periplasmic surface, rather than the L3 loop within the porin channel, might reduce the pore diameter of OmpC (Fig. 1g and Extended Data Fig. 3), supporting a porin-intrinsic mechanism of conductance control. To test this possibility experimentally, we generated an isogenic bacteria mutant by allelic exchange where the charged residues on the periplasmic surface of OmpC were replaced with alanines (E23A, K27A, D28A, E64A, D69A, D156A, D162A, D289A, K329A; termed mutant OmpC), which we expected to be unable to reduce porin permeability upon periplasmic acidification. In keeping with this hypothesis, mutant OmpC-expressing bacteria showed (compared to isogenic controls) the following: increased porin permeability, measured by 2NBDG uptake; decreased periplasmic acidification (measured using a reporter based on the pH sensitive fluorescent protein pHuji^52^), consistent with an increased porin-mediated proton leak; and, potentially as a consequence, a growth defect accentuated in media not supporting glycolysis (Fig. 1h and Extended Data Fig. 4).

**Extended Data Fig. 5:**
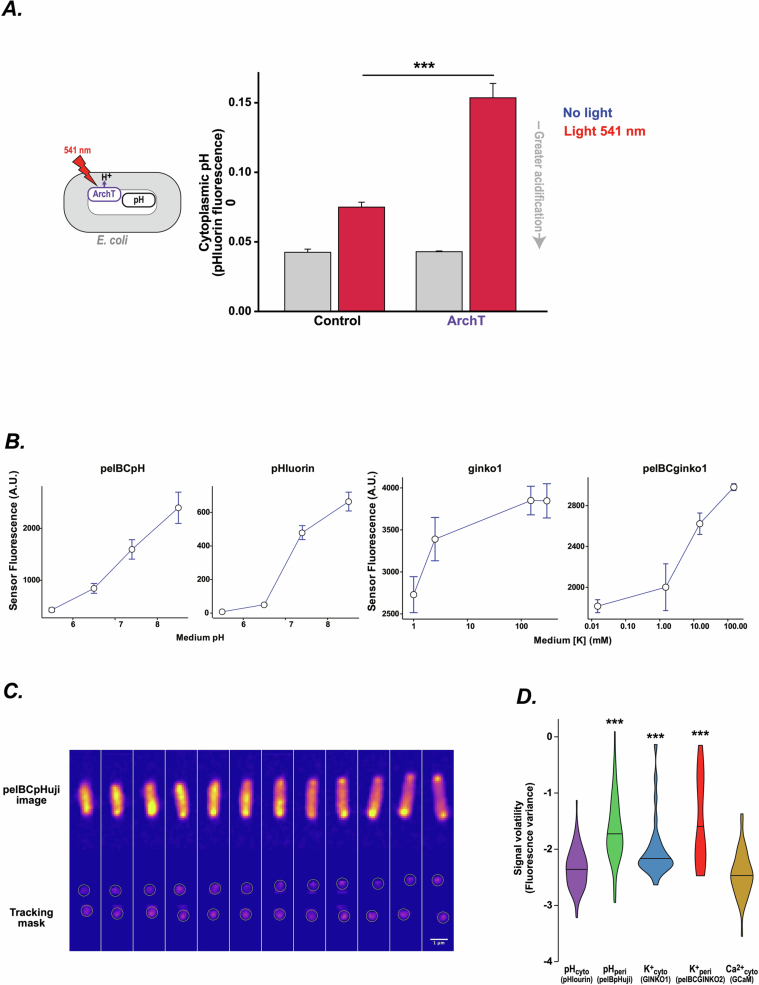
Optogenetic probes and fluorescent ion sensors expressed in *E. coli.* (**A**) Changes in cytosolic pH (assessed by the fluorescent reporter pHluorin) in E. coli expressing ArchT light-activation proton pump (right) or controls (left) in the absence (blue) or presence (red) of 561 nm illumination. Activation of Arch T results in significant alkalinisation of the cytosol as a consequence of pumping protons into the periplasm. ***p-value < 0.001 (**B**) Left. Calibration curve for cytoplasmic pHluorin and periplasmic pHuji. Cells were grown overnight and then seeded in the mother machine for microscopy observation (See methods for detailed protocol). The sensors were calibrated by flowing PBS + 1 g/L glucose + 250 μM CCCP and buffered at a range of pH (5.5, 6.5, 7.5, 8.5). Data shown (mean ± SEM) is representative of at least three independent experiments per condition. Right. Calibration curve for cytoplasmic GINKO1 and periplasmic-GINKO1. Cells were grown overnight and then seeded in the mother machine for microscopy observation (See methods for detailed protocol). The sensors were calibrated by flowing HEPES + 1 g/L glucose + 100 μM Valinomycin. The HEPES buffer then was balanced with NaCl or KCl in order to maintain the ionic strength constant at 150 mM. The KCl concentration was set at 150, 15, 1.5, and 0.015 mM. (**C**) Representative image of cell pole tracking algorithm for periplasmic measurements. The algorithm consisted in two steps. First, it segmented all the cells tracked the cells as a whole. Then, it segmented the top and bottom regions (associated with the periplasmic localisation) and tracked the fluorescence of those regions. (**D**) Fluorescence signal variance over time in individual wild type E. coli for each ion sensor detecting cytoplasmic pH (pH cyto; purple), periplasmic pH (pH peri; green), cytoplasmic potassium ions (K+ cyto; blue), periplasmic potassium ions (K+ peri; red), and cytoplasmic calcium ions (Ca2+cyto; gold). Data are representative of at least three independent experiments performed in at least triplicate, imaging at least 30 individual bacteria per condition. ***p-value < 0.001 (Student’s t-test relative to Ca2+ cyto signal). Source data

To experimentally examine whether directly altering periplasmic ions could influence porin permeability, we expressed the light-activated proton pump, ArchT^53,54^, in the inner membrane of WT *E. coli* to selectively acidify the periplasm (monitored by alkalinization of the cytoplasm; Extended Data Fig. 5) and monitored 2NBDG uptake using a microfluidic perfusion system that allows imaging of individual bacteria over time^55,56^. We found that, upon light exposure, ArchT-expressing *E. coli* (but not controls) showed reduced 2NBDG uptake (Fig. 1i,j and Supplementary Video 1), indicating that porin permeability is reduced upon periplasmic acidification.

### Temporal fluctuations in internal ion concentrations modulate porin permeability

**Fig. 2:**
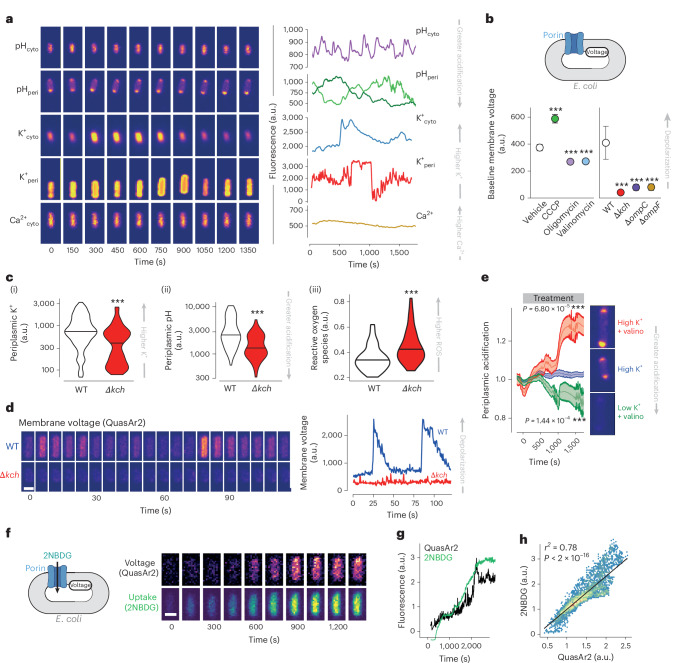
Fluctuations in periplasmic ions cause temporal changes in porin permeability. **a**, Representative images (left) and traces (right) over time of different individual bacteria (growing in M9 + 1 g l^−1^ casamino acids + 1 g l^−1^ glucose and 1 mM tryptophan media) imaged in the mother machine microfluidics platform expressing fluorescence sensors for cytoplasmic pH (pHcyto; pHluorin; purple), periplasmic pH (pHperi; pelB-pHuji; green), cytoplasmic K^+^ (K^+^cyto; Ginko1; blue), periplasmic K^+^ (K^+^peri; pelBC-ginko2; red) or cytoplasmic calcium (Ca^2+^cyto; GCaMp6f; yellow). Data are representative of at least three independent experiments with at least 50 bacterial cells per condition. **b**, Left: effect of CCCP (250 μM; green; *P* = 8.5 × 10^−8^), oligomycin (3 μM; violet; *P* = 0.00019), valinomycin (100 μM; light blue; *P* = 1.6 × 10^−8^) or vehicle alone (white) on baseline inner membrane voltage of individual bacteria (measured using the sensor QuasAr2 (ref. ^62^), which increases in fluorescence with membrane depolarization). Right: baseline inner membrane voltage in WT cells (white) or isogenic knockouts for *kch* (red), ompC (purple) or ompF (yellow). *N* = 3 biological replicates; all *P* < 2 × 10^−16^. *y* axes show QuasAr2 fluorescence in a.u. **c**, Single bacterial measurements of (i) periplasmic K^+^ (detected through changes in pelBC-GINKO2 fluorescence; *P* < 2 × 10^−16^), (ii) periplasmic pH (detected through changes in pelB-pHuji fluorescence; *P* = 6.4 × 10^−7^) and (iii) cytoplasmic reactive oxygen species (ROS) levels (detected through changes in HyPer7 fluorescence; *P* = 0.00037) in WT (white) or isogenic *kch* knockout strains (red) of *E. coli*. *y* axes show sensor fluorescence in a.u. **d**, Time-lapse montage and the trace of *E. coli* WT and *Δkch* cells expressing the membrane potential reporter QuasAr2. Representative images and traces of membrane voltage (measured by QuasAr2 fluorescence (a.u.) in WT *E. coli* (blue) or isogenic *kch* knockouts (red). **e**, Single-cell recordings of periplasmic pH over time (monitored by pelB-pHuji fluorescence) following treatment with 150 mM external K^+^ (0 mM external Na^+^) and 100 μM valinomycin (High K^+^ + valino; red), 150 mM external K^+^ (0 mM Na^+^) alone (High K^+^; blue) or 0 mM external K^+^ (150 mM external Na^+^) and 100 μM valinomycin (Low K^+^ + valino; green). Representative images after 1,500 s treatment. **f**,**g**, Representative images (**f**) and traces (**g**) of simultaneous recordings in single bacteria (WT *E. coli*) of membrane voltage (QuasAr2 fluorescence; black) and 2NBDG accumulation (green) over time. Data are representative of at least three independent experiments with at least 50 bacterial cells imaged per condition. **h**, Plot of membrane voltage and 2NBDG uptake in individual bacteria (*n* > 40) over time. Line shows the linear regression of QuasAr2 fluorescence against 2NBDG fluorescence. Data are representative of at least three independent experiments performed at least in triplicate per condition, imaging at least 50 individual bacteria per condition shown as mean ± s.e.m. (Student’s *t*-test) in **b**, or violin plots (Wilcoxon signed-rank test) in **c**. ***P < 0.001. In **b** and **e**, error bars and ribbon bands represent the s.e.m. All statistical tests were two-sided. Scale bars, 1 μm. Source data

We next explored how ion concentrations within bacteria might change over time by creating genetically encoded fluorescence sensors for cytoplasmic and periplasmic pH based on pHluorin and pHuji, respectively^52,57^ (which decrease in fluorescence with acidification) as well as sensors for cytoplasmic and periplasmic K^+^ (based on the potassium sensors GINKO1 and GINKO2, respectively^58^), and cytoplasmic Ca^2+^ (based on the calcium sensor CaM^59^), all of which increase in fluorescence with increasing ion concentration (Extended Data Fig. 5). Periplasmic localization of sensors was achieved by exploiting the *pelB* export system (as previously described^60,61^) with fluorescence from residual cytoplasmic protein excluded by image segmentation (Extended Data Fig. 5). Through single bacterial imaging, we observed dynamic changes in cytoplasmic and periplasmic H^+^ and K^+^ ions (but not cytoplasmic Ca^2+^) over time (Fig. 2a,b), with particular volatility seen in periplasmic H^+^ and K^+^ levels (Extended Data Fig. 5), raising the possibility of temporal regulation of porin activity through these ion fluctuations.

As changes in electrochemical gradients of H^+^ and K^+^ across a membrane would be expected to affect its voltage, we used a genetically encoded sensor (QuasAr2 (ref. ^62^)) to measure the bacterial inner membrane potential. We found that inner membrane voltage was influenced by changes in both periplasmic H^+^ and K^+^ as reducing periplasmic H^+^ (by treatment with CCCP) caused membrane depolarization; increasing periplasmic H^+^ (by inhibiting ATP synthase activity using oligomycin) caused hyperpolarization; and reducing periplasmic K^+^ (with valinomycin) caused hyperpolarization (Fig. 2b). We detected membrane hyperpolarization in deletion mutants of *ompC* or *ompF* (further supporting the concept of proton loss through porins^18^) and also in *kch* knockouts, indicating a major role for this ion channel in inner membrane ion conductance (Fig. 2b).

To further explore the role of Kch, we found that isogenic mutants had reduced periplasmic K^+^ levels (consistent with decreased cytosol-to-periplasmic K^+^ flux^63^), increased periplasmic H^+^ levels (in keeping with reduced porin-mediated proton loss) and increased reactive oxygen species, suggesting that Kch may act to dissipate excessive proton motive force across the inner membrane to reduce the production of oxygen free radicals, plausibly through the back reaction of Complex I of the ETC^64,65^ (Fig. 2c).

Through single bacterial imaging, we detected periodic membrane depolarizations (‘action potentials’) in WT bacteria, as previously described^66^, but not in *kch* knockout cells (Fig. 2d and Supplementary Video 2). As artificially altering periplasmic K^+^ levels in WT bacteria (by exposure to high or low potassium in the presence of valinomycin) leads to rapid changes in periplasmic H^+^ (Fig. 2e), the initiation of action potentials may be due to Kch-mediated K^+^ influx into the periplasm leading to porin opening and subsequent proton loss. This model predicts that depolarized bacteria will have increased porin permeability, and this is supported by our observation of a tight correlation between membrane voltage and 2NBDG uptake (Fig. 2f–h).

### Metabolic control of periplasmic ions regulates porin permeability

**Fig. 3:**
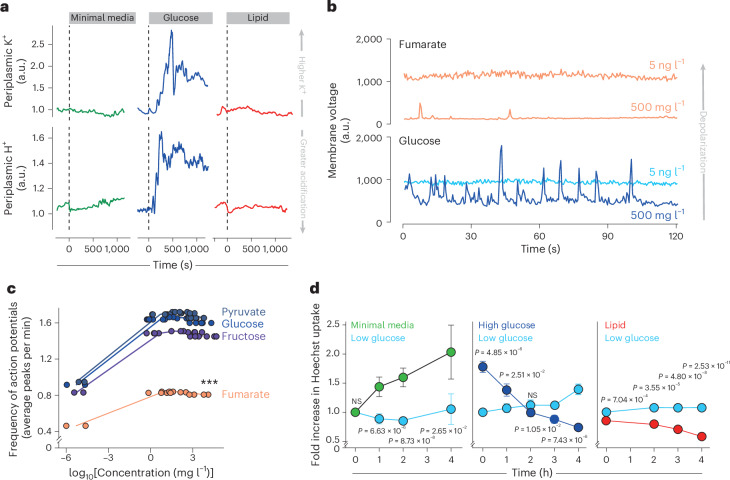
The impact of metabolism on periplasmic ions, membrane voltage and porin permeability. **a**, Representative traces of periplasmic K^+^ (top, measured by pelBC-GINKO2 fluorescence (a.u.)) and periplasmic H^+^ (bottom, measured by pelB-pHuji fluorescence (a.u.)) in individual bacteria when external media is changed to minimal media (M9, green), glucose media (M9 + 4 g l^−1^ glucose; blue) or lipid media (M9 + 0.014 g l^−1^ DPPC). **b**, Representative traces of changes in membrane voltage over time (monitored by QuasAr2 fluorescence (a.u.)) in individual bacteria exposed from low (5 ng l^−1^) to high (500 mg l^−1^) fumarate (orange) or glucose (blue). **c**, Frequency of action potentials (average peaks per min) for WT *E. coli* following exposure to media with different concentrations of fumarate (orange), fructose (purple), glucose (blue) or pyruvate (dark blue) as the only carbon source. Statistical analysis was performed using a generalized linear model (Methods; *P* = 1. 71 × 10^−9^ between fumarate and fructose). **d**, Porin permeability (detected through Hoechst accumulation measured by flow cytometry) of WT *E. coli* exposed to M9 media alone (minimal media; green), M9 media with low glucose (0.04 g l^−1^; light blue), high glucose (4 g l^−1^; dark blue) or lipid (0.014 g l^−1^ DPPC; red). Bacteria were incubated for 10 min at 37 °C with Hoechst at times indicated. Fluorescence normalized to low glucose fluorescence at 0 h. Data shown (mean ± s.e.m.) are representative of at least three independent experiments per condition, each performed at least in triplicate. ***P < 0.001 (two-sided Student’s *t*-test). Source data

**Extended Data Fig. 6:**
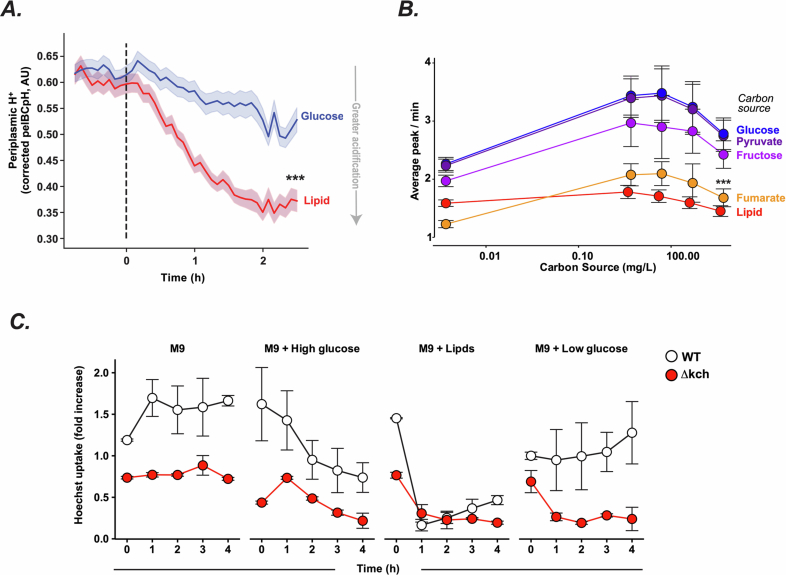
Effect of different carbon source metabolism on periplasmic pH, action potentials, and porin permeability in *E. coli.* (**A**) The relative change in pH (given by the background subtracted pHuji fluorescence signal in the cell pole compared to the cell body) in cells grown in lipid-based medium (red; n = 392) and cells grown in glucose-based medium (blue; n = 394). The black dashed line shows the time at which the media was switched from M9 to lipid-based or glucose-based medium. (**B**) Frequency of action potentials (average peaks/ minute) for wild-type E. coli following exposure to media with different concentrations of fumarate (orange), fructose (pink), glucose (blue), pyruvate (purple), or DPPC (red) as the only carbon source. (**C**) Porin permeability (detected through Hoechst accumulation measured by flow cytometry) of wild-type (white) and Δkch (red) E. coli in different carbon sources. The cells were exposed to M9 media alone (first panel), high glucose (4 g/L; second panel), M9 media with lipid (0.014 g/L DPPC; third panel) or low glucose (0.04 g/L; fourth panel). Cells were incubated for 10 minutes at 37 oC with Hoechst at times indicated. Fluorescence normalised to low glucose fluorescence at 0 h. The data shown (mean ± SEM) is representative of at least three independent experiments performed at least in triplicate per condition. Source data

We next explored the impact of metabolism on periplasmic ions and, consequently, porin permeability. Exposure of WT *E. coli* to glucose led to rapid and large fluctuations in periplasmic K^+^ and H^+^ levels, not seen in minimal media or media with lipids as the primary carbon source (Fig. 3a), the latter causing sustained periplasmic acidification (Extended Data Fig. 6). These results suggest that Kch opening might be related to the activity of the ETC and thus the metabolic state of bacteria, as previously proposed^63^. Consistent with this idea, we found that the frequency of action potentials increased with the quality and quantity of available carbon sources, with pyruvate and glucose (preferred substrates for Kreb’s cycle^55,67^), triggering the largest number of action potentials at any given concentration, and lipid and fumarate the least (Fig. 3b,c and Extended Data Fig. 6).

As expected from these results, the metabolic state of bacteria also affected porin permeability. We found a greater porin-mediated entry in bacteria exposed to minimal media than low glucose media, in keeping with higher porin opening under conditions of low periplasmic H^+^ (Fig. 3d). We also observed greater permeability in high glucose than in low glucose media and in low glucose than in lipid media, consistent with greater porin opening under conditions of higher periplasmic K^+^ (Fig. 3d), properties that were abolished in *Δkch* bacteria (Extended Data Fig. 6).

**Extended Data Fig. 7:**
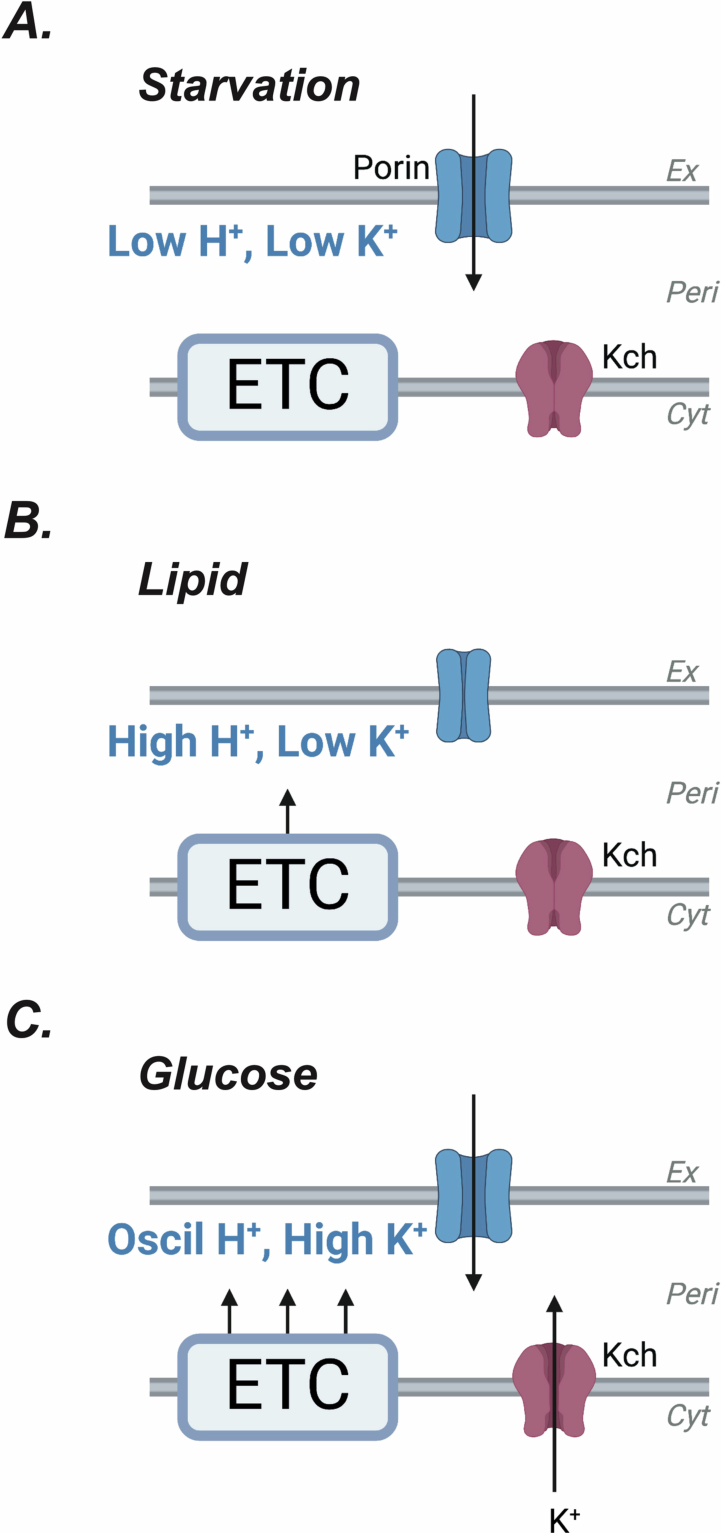
Model for metabolic control of porin regulation. Model explaining metabolic control of porin permeability. (**A**) During starvation, the permeability of porins (blue) is high due to low periplasmic H+ and K+ levels (resulting from minimal electron transport chain (ETC; grey) activity and no opening of Kch channels (pink), respectively). (**B**) Growth in lipids causes an increase in periplasmic H+ (through increased ETC activity) without activating Kch, resulting in low porin activity. (**C**) In contrast, growth in glucose media drives ETC activity and Kch channel opening, leading to fluctuating periplasmic H+ and high periplasmic K + , causing fluctuations also in porin permeability.

We, therefore, propose a model for metabolic control of porin regulation (Extended Data Fig. 7) whereby in minimal media without carbon source, porin permeability is high (due to low ETC activity and thus low periplasmic H^+^); during growth in lipid or other slowly used carbon sources, porin permeability is low due to high periplasmic H^+^ and low K^+^ levels, while during growth in rich media (such as glucose), porin permeability is high (due to high periplasmic K^+^ levels caused by Kch activation).

### Ionic control of porin permeability regulates antibiotic susceptibility

**Fig. 4:**
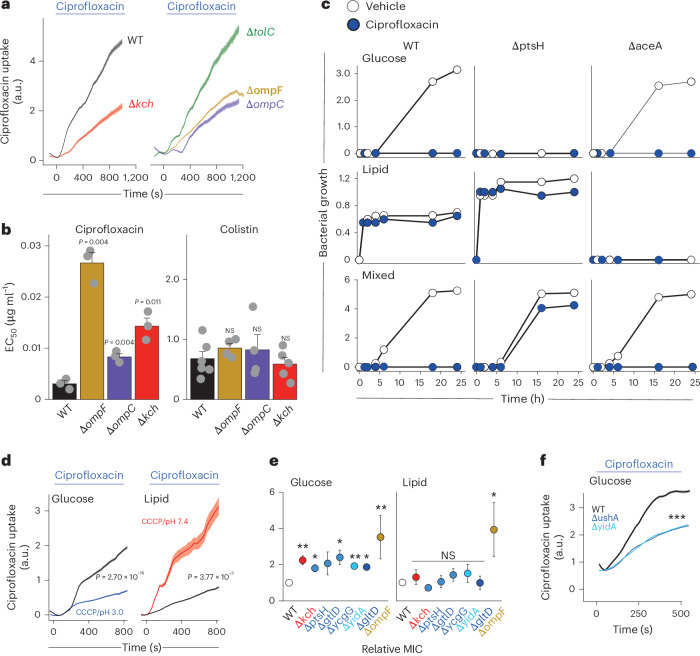
Metabolic control of porin permeability influences antibiotic resistance. **a**, Accumulation of ciprofloxacin in individual bacteria over time (monitored by fluorescence imaging of wild type *E. coli* using a microfluidics platform) assessed for WT bacteria (black), and isogenic knockouts for *kch* (red), the efflux pump component *tolC* (green), *ompF* (yellow) or *ompC* (purple). Ciprofloxacin fluorescence normalized to 0 s value. The data shown here (mean ± s.e.m.) are representative of at least three independent experiments per condition with at least 50 individual bacteria imaged each time. ***P < 0.001 (Student’s *t*-test). **b**, EC_50_ values for WT *E. coli* (black) and isogenic knockouts of *kch* (red), *ompF* (yellow) and *ompC* (purple) exposed to ciprofloxacin (left; *n* = 3) or colistin (right; *n* = 5). Data shown (mean ± s.e.m.) are representative of at least three independent experiments performed at least in triplicate per condition. NS, not significant (Student’s *t*-test). **c**, Bacterial growth (monitored by OD_650_) of WT *E. coli* (left) or isogenic knockouts of *ptsH* (middle) or *aceA* (right) in lipid media (0.014 gl^−1^ DPPC + 0.3 g of Bacto Casitone and 0.5 ml of Tyloxapol; middle row), glucose media (M9 + 1 g l^−1^ glucose; top row), mixed media (0.014 g l^−1^ DPPC + 0.3 g of Bacto Casitone and 0.5 ml of Tyloxapol + 1 g l^−1^ glucose; bottom row) in the presence of ciprofloxacin (0.025 μg ml^−1^; blue) or vehicle alone (white). **d**, Accumulation of ciprofloxacin in individual WT *E. coli* over time (monitored as in **a**) during exposure to media containing glucose (black) or glucose at pH 3.0 with CCCP (250 μM; blue) (left) or media containing lipid (black) or lipid at pH 7/4 with CCCP (250 μM; red; right). **e**, MICs of ciprofloxacin for WT (white) or isogenic knockouts for *kch* (red); *ptsH*, *gltD*, or *ycgG* (blue); yidA (light blue), ushA (dark blue), or *ompF* (yellow) grown in glucose (left) or lipid (right) media (MIC values normalized to WT for each media). Significant increases in relative MIC observed for *Δkch* (*P* = 0.0023), *ΔptsH* (*P* = 0.039), *ΔycgG* (*P* = 0.010), *Δ*yidA (*P* = 0.0029) *Δ*ushA (*P* = 0.014) and *ΔompF* (*P*= 0.0033) in glucose media but only *ΔompF* (*P* = 0.039) when grown in lipid media (Mann–Whitney *U*-test). **f**, Accumulation of ciprofloxacin within individual *E. coli* (monitored as in **a**, shown as ciprofloxacin fluorescence (a.u.)) grown in glucose media (M9 + 4 g l^−1^ glucose) comparing WT (black), *ushA* (dark blue; *P* = 2.26 × 10^−21^) or *yidA* (light blue; *P* = 9.61 × 10^−23^) isogenic knockout strains. Data shown (mean ± s.e.m.) are representative of at least three independent experiments per condition with at least 150 individual bacteria imaged each time. ***P < 0.001 (Student’s *t*-test). Source data

We next examined how ionic control of porin permeability might influence susceptibility to antibiotics, many of which show porin-mediated entry into bacteria^7^. Using single-cell fluorescence imaging, we observed reduced uptake of ciprofloxacin by OmpF, OmpC and Kch deletion mutants (confirming porin-dependent uptake and the expected action of Kch on porin conductance) but no impact of deleting the efflux pump component TolC^15^, indicating that, over the time course of these experiments, ciprofloxacin accumulation was only dependent on drug influx rather than efflux (Fig. 4a).

**Extended Data Fig. 8:**
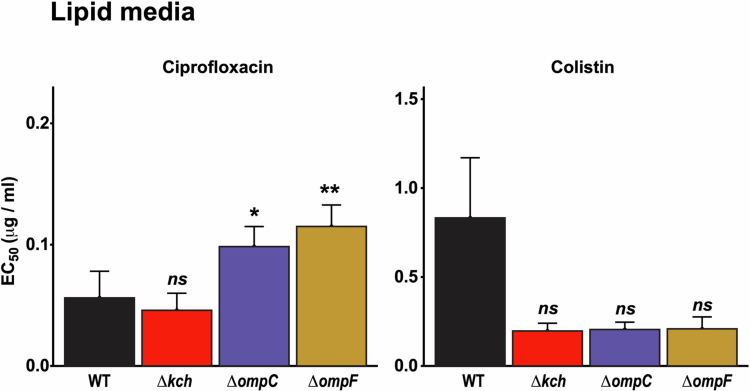
Effect of gene deletions on antibiotic susceptibility for *E. coli* grown in lipid media. EC50 values for wild-type E. coli (WT; black) and isogenic knockouts of kch (red), ompC (purple) and ompF (yellow) exposed to ciprofloxacin (left) or colistin (right) when growing in M9 + Lipids media. Data shown (mean ± SEM) is representative of at least three independent experiments performed at least in triplicate per condition. ns not significant, *p < 0.05, ** p < 0.01, *** p < 0.001 (Student’s t-test). Source data

We found that, when grown in glucose media, *ompF*, *ompC* and *kch* knockout mutants showed increased resistance to ciprofloxacin (but not to colistin, an antibiotic that is porin independent^3,68,69^), suggesting functional consequences of altered porin permeability (Fig. 4b). We, therefore, wondered whether the recognized effects of different carbon sources on antibiotic susceptibility^67,70–74^ could be mediated through changes in porin permeability. We first confirmed that ciprofloxacin susceptibility was greater for *E. coli* grown in glucose compared to lipid media (Fig. 4c). To understand the potential effects of different carbon sources on antibiotic stability, solubility or availability, we examined the behaviour of mutants unable to grow in glucose (*ΔptsH*) or in lipid (*ΔaceA*) media. When grown in mixed media, WT bacteria and *ΔaceA* mutants showed susceptibility to ciprofloxacin, while *ΔptsH* mutants were found to be resistant (Fig. 4c), implying that utilization of glucose leads to antibiotic susceptibility. As expected, *kch* knockout bacteria were equally resistant to isogenic wild type controls when grown in lipid media (Extended Data Fig. 8), conditions associated with no kch activity (Fig. 4a).

We observed that ciprofloxacin uptake was greater in bacteria grown in glucose than in lipid media but could be reduced in glucose media by increasing periplasmic H^+^ (by exposure to CCCP and pH 3 external media) and could be enhanced in lipid media by decreasing periplasmic H^+^ (by exposure to CCCP and pH 7.4 external media) (Fig. 4d), findings that indicate that ionic regulation of porin function may mediate metabolic control of antibiotic susceptibility.

As mutations in genes involved in central metabolism have been shown to cause antibiotic resistance in clinical and experimental settings^71^, we wondered whether their effect might be mediated, at least in part, through changes in porin permeability. Deletion mutants for several genes previously implicated in drug resistance^71^ showed increased ciprofloxacin resistance in glucose but not in lipid media (Fig. 4e) and displayed reduced ciprofloxacin uptake when grown in glucose (Fig. 4f), suggesting that these mutations prevent porin opening triggered by high metabolic activity.

### Summary

Our findings, therefore, show that the permeability of porins in *E. coli*, and potentially other Gram-negative bacteria, can be dynamically regulated through changes in periplasmic H^+^ and K^+^, which in turn are influenced by the metabolic state of the cell via the action of the ETC and the Kch potassium channel. Although our initial results were based on experiments with valinomycin and CCCP and therefore potentially influenced by indirect effects of these compounds on bacterial physiology, a role for periplasmic pH and K+ in the control of porin permeability is supported by our orthogonal genetic and optogenetic approaches. Ionic control of porin permeability thus provides a mechanism by which nutrient uptake is linked to substrate utilization while preserving the periplasmic proton motive force. Our results also explain the recognized reduction in antibiotic susceptibility when bacteria are grown in lipid media and the impact of mutations in central metabolism genes on antibiotic resistance. Our results suggest that therapeutic activation of Kch may enhance antibiotic accumulation within bacteria and could improve killing under conditions such as during intracellular infection^75,76^, where lipids are used as the primary carbon source.

## Methods

### Bacterial strains and growth conditions

#### Bacterial strains

The following strains were from the Keio collection^1^ (Horizon Discovery): *E. coli* K12 BW25113 WT, *ΔompF*, *ΔompC*, *ΔompG*, *ΔnmpC*, *ΔphoE*, *Δkch*, *ΔclcB*, *ΔptsH*, *ΔaceA*, *ΔgtlD*, *ΔycgG*, *ΔyidA*, *ΔushA* and *ΔtolC*. The long-term stock was stored in 20% glycerol at −80 °C. Bacterial samples were stroked monthly in Luria Broth (LB) + agar with or without antibiotics in which kanamycin was used for selection at 50 µg ml^−1^. Single colonies were resuspended in a 50 ml falcon tube with 10 ml fresh media as indicated. These cultures were incubated in glass test tubes with a breathable lid in a shaking incubator at 37 °C in an orbital shaker at 300 r.p.m.

#### Media composition

The super-optimal broth (SOB) or minimal medium 9 (M9) was used for each experiment. The SOB was prepared following a premix recipe (Fromedium, SOB01CFG). To prepare the minimal medium, we adapted the recipe described by Kotte^2^: The medium contained the following components: base salt solution (211 mM Na_2_HPO_4_, 110 mM KH_2_PO_4_, 42.8 mM NaCl, 56.7 mM (NH_4_)_2_SO_4_, autoclaved and prepared by the scientific facilities at the MRC Laboratory of Molecular Biology (LMB)), 10 ml of trace elements (final concentration: 0.63 mM ZnSO_4_, 0.7 mM CuCl_2_, 0.71 mM MnSO4, 0.76 mM CoCl_2_, autoclaved), 0.1 ml 1 M CaCl_2_ solution (autoclaved, prepared by the scientific facilities at LMB) for a final concentration of 0.1 mM, 1 ml 1 M MgSO_4_ solution (autoclaved, prepared by the scientific facilities at LMB) for a final concentration of 1 mM, 2 ml of 500× thiamine solution (1.4 mM in ultrapure water from Milli-Q, Millipore and filter sterilized) and 0.6 ml 0.1 M FeCl_3_ solution (filter sterilized). The final volume was adjusted to 1 l with ultrapure water (Milli-Q, Millipore) lab water system (Milli-Q Advantage-10, Millipore). For each batch, the medium was filtered through a 0.22 µm Millipore Stericup and split into 500 ml bottles. Before each experiment, the medium was supplemented with 1 g l^−1^ casamino acids, 1 mM tryptophan and 0.5 g l^−1^ glucose unless the contrary was specified. Tryptophan stock was prepared at 50 mM in water from powder (Sigma Aldrich) and kept at 4 °C. Casamino acids (VWR Life Science) stock was prepared at 100 g l^−1^ in MiliQ water and kept at 4 °C.

#### Carbon sources

The carbon sources used in this work (glucose, fructose, acetate, fumarate, pyruvate) were acquired from Sigma-Aldrich in powder form. Then, stock solutions were prepared at a 100 g l^−1^ concentration in Milli-Q water. After adjusting the pH to 7.0, 100 ml aliquots were filtrated with a 0.22 µm syringe filter. For the lipid media preparation, we used 14 mg l^−1^ 1,2-dipalmitoylphosphatidylcholine (DPPC), combined with 0.3 g l^−1^ casitone and 0.05% Tyloxapol to allow proper dilution.

### Plasmid design and construction

The primers were designed with the Primer3 algorithm available through the Benchling platform^3^. The DNA material was amplified by PCR with the PrimeStart HS polymerase kit (Takara), following the manufacturer protocol and adjusting the annealing temperature to the suggested by the Benchling cloning algorithm. The amplification cycles were repeated 30 times using a C1000 Touch Thermal Cycler (BioRad).

The resulting DNA fragments were ligated with the Gibson assembly kit. The concentration of the DNA templates was estimated with UV absorption using a NanoDrop 3300 (ThermoFisher), and the NEBiocalculator helped us estimate the required volumes for the Gibson assembly. All assemblies were carried out for 1 h at 50 °C using the same thermocycler as the PCR amplification. Bacterial transformation of the assembled sequence was carried out through heat shock using *E. coli* DH5α as an intermediate strain. Isolated plasmids were stored at −20 °C in Qiagen Elution Buffer.

#### pBAD_pelBCpHuji assembly

The periplasmic pH sensor was based on the arabinose-inducible pBAD TOPO cloning vector system. This backbone was amplified with primers listed in Supplementary Table 1. To this backbone, we added the pectate lyase B (pelB) leader sequence. The pelB export signal sequence has proven to be successful in delivering recombinant proteins to the periplasm of *E. coli*^4,5^. This signalling sequence consists of 22 amino acids placed at the start codon. The pelBC fragment was amplified with primers listed in Supplementary Table 1. Then, we inserted the pH sensor. To measure the periplasmic pH accurately, we looked for a sensor with a wide dynamic range because the periplasmic pH could be up to 2 units lower than the cytoplasm, which means a pH 5 (ref. ^6,7^). As the flurescence of the pH sensor pHluorin collapses below pH 6, we opted for pHuji with a working range between 5 and 9 (ref. ^8^). For the amplification of the pHuji fragment, we used primers listed in Supplementary Table 1. During the assembly process, we used *E. coli* DH5α, and after validating the sequence, we transferred the construct to an *E. coli* K12 BW25113 WT strain.

#### pBAD_pelBCginko2 assembly

We use the pelBC-pHuji plasmid to extract the backbone with the periplasmic export signal pelB. As GINKO1 is derived from green fluorescent protein, which is not stable in the periplasm, we designed a new variant based on super folding yellow fluorescent protein (sYFP) for improved periplasmic stability. Thus, we amplified the potassium-binding region of GINKO1 with primers listed in Supplementary Table 1 and inserted it between the 171 and 172 amino acids residues of sYFP. The sYFP was amplified with primers listed in Supplementary Table 1. This strategy was designed following the GINKO1 architecture. The final construct was amplified inside *E. coli* DH5α, and after validating the sequence, we transferred the construct to the *E. coli* K12 BW25113 strain.

#### pBAD_ArchT assembly

We used the pLenti-CaMKIIa-eArch 3.0-EYFP plasmid for the ArchT amplification^9,10^. For the PCR amplification, primers listed in Supplementary Table 1 were used. The backbone was derived from the pBAD_TOPO plasmid via digestion with the NcoI and PmeI restriction enzymes. Then, both fragments were ligated with the Gibson Assembly Kit.

#### Other genetic material

The plasmids pBAD_GINKO1 (Addgene code: 113111), pBAD_QuasAr2 (Addgene code: 64134) and GCaM-mRuby (also called pJMK0004, addgene code: 98920) were obtained via Addgene. The pBAD_pHluorin plasmid was developed in previous work^10,11^.

### Mutant generation

*E. coli* DH5a was used as a maintenance strain for all cloning steps. The *ompC* mutant allele vector (pBAD backbone) was constructed using a combination of traditional digestion/ligation cloning and Gibson assembly of mutant allele and chloramphenicol resistance cassettes, respectively.

Briefly, the *ompC* mutant allele (ompC_mut) was synthesized by Thermo Life Technologies with the following amino acid changes from the source sequence (ompC_source): E23A, K27A, D28A, E64A, D69A, D156A, D162A, D289A, K329A. The ompC_mut sequence was then amplified using primers listed in Supplementary Table 1 and cloned into the pBAD vector using the restriction site pair NcoI and PmeI to generate pBAD-ompC_mut. A chloramphenicol resistance cassette was amplified from the plasmid pT2SC using primers listed in Supplementary Table 1 and was subsequently introduced to the pBAD vector using Gibson assembly at the PmeI site, generating the final vector pBAD-ompC_mut-ChlR. An ompC_mut-ChlR fragment was then PCR amplified from pBAD-ompC_mut-ChlR with *E. coli* chromosomal homology regions using primers listed in Supplementary Table 1. The *ompC* mutant was then generated using *E. coli* BW25113 and the ompC_mut-ChlR fragment via λ Red recombinase-mediated allelic replacement as described in ref. ^77^. Mutants were confirmed by PCR and Sanger sequencing^12^.

### Permeability quantification using flow cytometry

#### Tracer uptake

The method for permeability estimation in bacteria was derived from refs. ^38,44,78^. Briefly, bacteria cultures were grown overnight at 37 °C in minimal medium M9, supplemented with casamino acids at 1 g l^−1^, tryptophan at 1 mM and glucose at 0.5 g l^−1^. The next day, the source cultures were diluted to an OD ≈ 0.05 in 25 ml fresh medium and placed in a 250 ml conical flask. Glucose was not added to the medium for 2NBDG experiments. When the turbidity reached an OD of 0.1–0.25, the bacteria were split in a 96-well U-shape plate with 180 μl per well. Here, 10 μl of the treatment solution was added, and the plate was sealed with gas-permeable film (4titude, PCR0548) and returned to the incubator. After 30 min, 10 μl of the fluorescent tracer from the reservoir was added. Then, the concentration of the fluorescent tracer in the reservoir was diluted so we could keep the final volume constant.

After adding the fluorescent probe, the plate was returned to incubation for the specified time. Next, 200 μl was transferred to a V-bottom 96-well plate (Costar), and it was centrifuged 5 min at 3,200 × *g* (Eppendorf 5810 R, rotor S-4-104) and washed twice with PBS. Finally, samples were fixed and resuspended in PBS + 4% formaldehyde, stored at 4 °C and analysed after 16 h. Then, we used the iCyt Eclipse (Sony) flow cytometer to read the fluorescence from all the samples. This device is equipped with 488 nm, 561 nm and 642 nm laser sources, along with Hoechst and FITC filter sets.

For the data analysis, background fluorescence was subtracted, and the cell size effect was corrected by dividing tracer fluorescence by the forward scatter signal^79^. For each experiment, the signal was normalized to WT strain or vehicle treatment.

#### Tracer uptake in different carbon sources

For the permeability estimation in different carbon sources, bacteria were grown as described before. When the cultures’ turbidity reached an OD of 0.1–0.25, the cultures were spun down for 5 min at 3,200 × *g* (Eppendorf 5810 R, rotor S-4-104), washed with M9 and resuspended in 15 ml fresh medium. The different medium compositions were M9 + casamino acids at 1 g l^−1^, tryptophan at 1 mM and glucose at 0.5 g l^−1^; M9 + 4 g l^−1^ (high) or 0.04 g l^−1^ (low) glucose; or M9 only (no carbon source). Next, the cultures were transferred to a 37 °C water bath, and 400 μl samples were taken at the indicated time points. A 400 μl sample was transferred to an Eppendorf tube, and 4 μl Hoechst (10 mg ml^−1^) was added to a final concentration of 10 μg ml^−1^. The tubes were inverted 3 times and incubated at 37 °C for 10 min. The samples were centrifuged for 30 s at maximum speed at 4 °C (Eppendorf centrifuge 5415 R), washed once and resuspended in 400 μl ice-cold PBS. Data analysis was performed as described in the previous section.

### Microfluidics imaging

#### Mother machine design and assembly

We used the mother machine design described in refs. ^55,80^. The microfluidic device was fabricated from an epoxy master template courtesy of Dr Jehangir Cama (University of Cambridge/University of Exeter, UK). The mother machine consists of a feed trench (50 µm × 100 µm × 30 mm) with many channels (1.4 µm × 1.4 µm × 25 µm) attached perpendicular to the trench.

For each batch of 12 chips, 50 ml of poly(dimethyl siloxane) (PDMS) was mixed with a 5 ml curing agent (10:1) with vigorous stirring. The bubbles formed during the mixing were removed by vacuum degassing for 20 min until all air bubbles disappeared. Then, the gel was poured onto the master template and baked at 100 °C for 1 h. Subsequently, the chip was cut out around the wafer and prepared for bonding with the cover slide. Holes for inlets and outlets were punched using a sharpened 0.6 mm biopsy puncher (from Fisher Scientific), and the chip was cleaned with Scotch tape and 2-propanol. After drying all the excess isopropanol, the coverslip and PDMS with the features upwards were exposed to air plasma in a vacuum for 20 s at 0.4 mbar oxygen (PlasmaPrep2, Gala Instrumente). This process activates the PDMS, which was put on a glass coverslip (24 mm × 50 mm, thickness 0.17 ± 0.005 mm, Carl Roth). Finally, the chips were incubated overnight at 65 °C.

Once the chip was ready, we flushed the chamber with 2-iso-propanol and sonicated the chip for 30 min to remove all the debris (FB50, Fisher-Scientific). Then, we coated the internal chip surface to facilitate bacterial attachment with a passivation buffer (containing herring sperm DNA and bovine serum albumin). The passivation buffer contained 10 mg ml^−1^ of bovine serum albumin and 10 mg ml^−1^ of salmon sperm in a 3:1 ratio^55^. The process was carried out overnight. Finally, the chips were stored in a dry cupboard for a week until required.

#### Trapping cells in the mother machine

The bacteria preparation started with an overnight culture in the specified medium. Before injecting the cells into the microchip, 2 ml of cells was washed by centrifugation (Eppendorf 5810 R) and resuspended in 2 ml of fresh medium. These cells were incubated for 10 min after and then centrifuged and concentrated to 100 µl. This high-density culture was injected into the microfluidic chip and set in a plate shaker at 37 °C for 10 min at 300 r.p.m. (Grant-bio, PHMP). Once the cells were trapped in the channels, the chip was connected with an inlet and outlet tubing (Tygon ND-100-80 Medical Tubing, 0.5 mm ID, 1.52 mm OD). Next, we flushed the chip with plain M9 for 10 min so that any cells remaining in the main channel were removed.

#### 2NBDG uptake

2NBDG permeability was estimated by measuring the increase of fluorescent signal of *E. coli* cells trapped in the mother machine side channels. The recording set-up of the 2NBDG videos was done in a Nikon-N STORM, with a CCD camera (Andor-DU-897) and a ×100/1.49NA lens. The microscope had a closed environment chamber to maintain the temperature constant at 37 °C. For illumination, we used a 488 nm and 561 nm laser light (Agilent Technologies, MLC-400B). The 2NBDG fluorescence signal was captured with a dichroic mirror 525/50 (TRF49909), and for the 561 nm laser excitation experiments, we used a quad-band filter (97335) in combination with a 525/50 nm filter.

#### Ciprofloxacin uptake

Cells were grown overnight in M9 + 1 g l^−1^ casamino acids + 0.5 g l^−1^ glucose + 1 mM tryptophan. The next day, cells were trapped in the mother machine as described in the previous section. Then, cells were resuscitated for 1–2 h until growth started. Then, 12.5 µg ml^−1^ of ciprofloxacin was added to the medium and started flowing into the chamber. Ciprofloxacin uptake was measured in a Zeiss 780 microscope, equipped with a UV light source (DPSS 355 nm 60 mW, Coherent), which allowed ciprofloxacin imaging. This instrument was also equipped with a thermal isolation box to keep the temperature constant at 37 °C. The imaging was done with a ×63/1.4NA oil lens. Ciprofloxacin fluorescence detection was done with a modified DAPI filter setting (435/65 nm)^15,29^.

#### Ion sensor calibration

Cell trapping was done as described above. Cells were then resuscitated with M9 + 0.5 g l^−1^ glucose + 1 g l^−1^ casamino acids + 1 mM tryptophan for 90 min. Then, for pH calibrations, we switched the medium to M9 + 1 g l^−1^ glucose for 30 min and finally to PBS calibrated to a determinate pH with 1 g l^−1^ glucose. The pH selected points were 8.5 and 5.5. The same procedure was done by adding 250 µM CCCP to the medium to permeabilize the cells. In the case of the potassium calibration, we used a medium HEPES pH 7.4 with complementary concentrations of KCl and NaCl to keep the osmolarity constant at 150 mM. To permeabilize cells to the extracellular potassium, we treated cells with 100 µM valinomycin. The flowing conditions were kept at 0.15 ml h^−1^, and during the switch, we increased the speed to 1 ml h^−1^ for 15 min. The illumination conditions for each sensor were the following: pelBCpHuji (excitation (Ex), 561 nm; emission (Em), 620/60 nm), pHluorin (Ex, 488 nm; Em, 525/50), ginko1 (Ex, 488 nm; Em, 525/50), pelBCginko2 (Ex, 488 nm; Em, 525/50), GCaMp6f (Ex, 488 nm; Em, 525/50) and QuasAr2 (Ex, 561 nm; Em, 650/long pass (LP) filter).

#### Ion sensor fluctuations

To observe the ion oscillations, we resuscitated cells with M9 + 0.5 g l^−1^ glucose + 1 g l^−1^ casamino acids + 1 mM tryptophan. The flow and temperature were kept constant at 0.150 ml h^−1^ and 37 °C. After 90 min, we started the recording process, which lasted until cells started dividing. For the data analysis, only 45 min before starting cell division was considered.

#### Carbon source switches

After trapping the cells into the chip, cells were washed with a plain M9 medium. Once the main channel was clear, the flow was kept constant at 0.15 ml h^−1^ for 30 min. At this point, we changed the syringe to the required carbon source and increased the flow to 1 ml h^−1^. After letting the medium flow for 2 min, we started the recording.

### Agarose pad experiments

Cells were grown overnight in SOB + 100 µg ml^−1^ ampicillin supplemented with 0.002 g l^−1^ arabinose and 20 µM retinal if required. The next day, cells were washed with M9, supplemented with the specified carbon source and resuspended for 10 min. Then, 2 µl was transferred to the agar pad. Before starting the recording, pads were left to dry for 5 min.

Agar pads were prepared on the same day of the experiment and discarded afterward. The gel was composed of a 10 ml M9 medium with 1.5 % (wt/vol) low-melting agarose and the specified carbon source. We then proceeded to dissolve the agarose by heating. Once the mixture was entirely homogeneous, the liquid was split into 3 ml portions onto a 35 mm petri dish (Falcon, ref: 353001). These plates were left to dry for 30 min. Once the agar solidified, we used a 6 mm biopsy puncher (Uni-Core, Harris UK) to cut out a single-use disk. We transferred 1 µl of cells on top of these agar disks, and after the drop was absorbed, the pad was moved onto a CELLview dish with glass bottom (627870, Greiner). Recording started immediately.

### Minimal inhibitory concentration and EC_50_ measurements

Minimal inhibitory concentrations (MICs) and effective concentration 50 (EC_50_), defined here as the drug concentration that induces a 50% maximal inhibitory effect on cell growth^81^, were determined for *E. coli* according to the Clinical and Laboratory Standards Institute method M07-A9 (ref. ^82^). Briefly, *E. coli* strains were grown to an optical density (A600 nm) of 0.2–0.3 in liquid culture, and 1 × 10^5^ bacteria were added to each well of 96-well plates containing serial dilutions of the antibiotic in triplicate wells per condition and incubated at 37 °C until growth was seen in the control wells. Then, the turbidity absorbance at 600 nm was measured with a ClarioStart (BGM LABTECH). Finally, the data were fitted to a dose–response model using a four-parameter logistic regression equation^83^. For the experiments in different carbon sources, cultures were grown in M9 + 4 g l^−1^ glucose or M9 supplemented with lipids (see ‘Tracer uptake in different carbon sources’ for full details).

### Growth curves in the presence of ciprofloxacin

Individual *E. coli* colonies (WT, *ΔptsH* and *ΔaceA*) were resuscitated in 10 ml of M9 media supplemented with 1 g l^−1^ glucose, M9 + lipid media (see ‘Bacterial strains and growth conditions’ for detailed composition), or mixed medium into 50 ml Falcon tubes. These cultures were incubated overnight at 37 °C with shaking. Note that the cultures grown in lipid media were incubated for slightly longer time due to their slower growth rate in this carbon source. Next, the cultures were grown to an optical density (OD_650_) between 0.2 and 0.3. Finally, these cultures were diluted at 1:1,000, and McFarland unit readings were taken at 1, 2, 4, 8, 16 and 24 h.

### Plasmid design and construction

The plasmids presented in this work were constructed using Gibson assembly^84^. The plasmids pBAD_QuasAr2 (QuasAr2, 64134), pBAD_Ginko1(ginko1, 113111) and pKL004 (GCaMp6f, 98920), were obtained from the Addgene database^52,58,59,62^. The plasmids for the expression of the pH sensor pHluorin were developed in previous work^85^. Finally, the plasmids pBAD_ArchT, pBAD_pelBCpHuji and pBAD_pelBCginko2 were developed specifically for this work. Further information on plasmid construction can be found in Supplementary Table 1.

### Molecular dynamic simulations

All simulations were performed by GROMACS v.4.6 (www.gromacs.org) with CHARMM36^86^ force fields for 100 ns. The trimeric OmpC (PDB ID: 2J1N, resolution 2.0 Å, with 346 amino acid residues^87^) and OmpF (PDB ID: 2OMF, resolution 2.40 Å, with 340 amino acid residues^88^) were used in this experiment. Eight independent simulations were performed, four for OmpC and four for OmpF. All systems were prepared using the CHARMM-GUI web interface. First, OmpC simulation was run at physiological pH. The second OmpC simulation was run with protonated L3 loop residues (D99, D105, D113, D118, E119) and D315 on the beta-barrel wall behind the L3 loop. The third simulation was run with protonated periplasmic side chains (E2, D6, D12, E43, D48, D84, D135, D141, D187, E189, D228, D268). Protonation state was calculated using the PROPKA3 (ref. ^89^). The last simulation was run with protonated L3 loop/periplasmic residues. The same hypothesis was applied to OmpF. The first simulation was run at physiological pH. The second OmpF simulation was run with protonated L3 loop residues (D107, D113, E117, E121, D126, D127) and D312 on the beta-barrel wall behind the L3 loop. The third simulation was run with protonated periplasmic side chains (E2, D6, D12, E48, D54, D92, D149, E183, D221). The orientation of proteins in membranes server was used for the orientation and position of the protein in the membrane, and each system was embedded in a pre-equilibrated neutral zwitterionic lipid phosphatidylcholine (POPC) bilayer. All the simulations were achieved at constant pressure (1 atm) and temperature (300 K). The results were analysed by locally written code. This was calculated from the output of multiple sampled conformers. Molecular graphic images were prepared using pymol 3.1.

The porin diameter was measured using the HOLE (v.2.2)^90^ software. More precisely, the diameter was determined using the block averaging method. The trajectory data were divided into blocks of frames, and the pore size was calculated for each block. The mean and standard deviation of the pore size across all blocks were computed. The standard deviation of the mean over blocks was used as a measure of the statistical error.

### Membrane voltage spike frequency

The QuasAr2 spike count was modelled with the glmmTMB^91^ R package (v.1.0.0). The model considers the concentration of the carbon sources (in log scale) and corrects for the non-spiking cells. The model parameters can be found in Supplementary Table 2.

### Statistical analysis

Statistical analyses were performed using R software (version 3.4.0). Data are presented as mean ± standard error of the mean (s.e.m.) from at least three independent experiments performed in triplicate unless otherwise stated. For flow cytometry experiments, background fluorescence was subtracted, and signals were normalized to WT strain or vehicle treatment controls. Statistical significance between two groups was determined using two-tailed Student’s *t*-test. For non-parametric data, Wilcoxon signed-rank test was applied. Multiple comparisons were corrected using Bonferroni adjustment where appropriate. The frequency of membrane voltage action potentials was analysed using generalized linear mixed models with the glmmTMB package, accounting for concentration effects in log scale and correcting for non-spiking cells. Dose–response curves for antibiotic susceptibility were fitted using four-parameter logistic regression. Statistical significance was defined as *P* < 0.05, with significance levels indicated as *P < 0.05, **P < 0.01, ***P < 0.001.

### Reporting summary

Further information on research design is available in the Nature Portfolio Reporting Summary linked to this article.

## Supplementary information

Reporting Summary

Supplementary Table 1 DNA primers used in this work.

Supplementary Table 2 Statistical parameters for generalized linear mixed model analysis of membrane voltage action potential frequency in response to carbon source concentration.

Supplementary Video 1 Representative single-cell fluorescence imaging of *E. coli* grown in a microfluidic perfusion system measuring 2NBDG accumulation over time in wild-type bacteria expressing empty vector (WT) or expressing the light-activated proton pump ArchT in the inner membrane in the presence of 561 nm light exposure.

Supplementary Video 2 Representative single-cell fluorescence imaging of *E. coli* WT and *Δkch* cells expressing the membrane potential reporter QuasAr2. Membrane depolarization causes increased fluorescence.

## Source data

Source Data Fig. 1 All data presented in Fig. 1.

Source Data Fig. 2 All data presented in Fig. 2.

Source Data Fig. 3 All data presented in Fig. 3.

Source Data Fig. 4 All data presented in Fig. 4.

Source Data Extended Data Fig. 1 All data presented in Extended Data Fig. 1.

Source Data Extended Data Fig. 5 All data presented in Extended Data Fig. 5.

Source Data Extended Data Fig. 6 All data presented in Extended Data Fig. 6.

Source Data Extended Data Fig. 8 All data presented in Extended Data Fig. 8.

## Data Availability

Protein structures and protein sequences used in this work are publicly available. Source data are provided with this paper.
